# Medication-Related Problems Identified Through Continuous Medication Monitoring

**DOI:** 10.3390/pharmacy6030086

**Published:** 2018-08-20

**Authors:** Amber M. Goedken, Sharon Huang, Randal P. McDonough, Michael J. Deninger, William R. Doucette

**Affiliations:** 1 College of Pharmacy, University of Iowa, Iowa City, IA 52242, USA; sharon-huang@uiowa.edu (S.H.); william-doucette@uiowa.edu (W.R.D.); 2 Towncrest Pharmacy, Iowa City, IA 52240, USA; mcdonough@towncrest.com (R.P.M.); deninger@towncrest.com (M.J.D.)

**Keywords:** community pharmacy, medication-related problems, medication monitoring, refill medication

## Abstract

Community pharmacists performing Continuous Medication Monitoring (CoMM) systematically monitor each new prescription and refill dispensed for medication-related problems. The objectives for this study were to describe medication-related problems identified through CoMM and drug classes involved in problems. This 12-month pilot study used dispensing and clinical records from a single independent U.S. community pharmacy. Clinical records contain medication-related problems documented by the pharmacists. Problems identified for patients filling at least one prescription at the pharmacy and having at least one medication-related problem during the study period were included. A total of 8439 medication-related problems were identified for 1566 patients, an average of 5.4 problems per patient. Over 63% of problems were nonadherence. The drug class most often involved in problems was the central nervous system and analgesic class. Community pharmacists performing CoMM identified medication-related problems that might otherwise have gone undetected.

## 1. Introduction

Medication-related problems may develop during treatment. One-quarter of ambulatory patients taking a new medication experienced an adverse drug event [1]. Nonadherence to medication also is well-documented [2]. Prospective drug utilization review (pro-DUR) by pharmacists is designed to detect medication-related problems by reviewing patients’ medication profiles for appropriateness prior to dispensing medication. There are some requirements for pharmacists to perform pro-DUR in the U.S., but the requirements are not consistent across the nation. Subsequently, pro-DUR may not be performed for refills or for all patients. While pharmacists are expected to address problems identified during pro-DUR, documentation of problems is not usually required.

Recognizing the shortcomings of pro-DUR, at least one community pharmacy in the U.S. engages in Continuous Medication Monitoring (CoMM), previously described in greater detail [3]. In short, CoMM goes beyond pro-DUR by systematically assessing each new prescription and refill dispensed for problems, collecting additional information from patients, taking action to resolve identified problems, and documenting problems and actions taken by the pharmacist. The monitoring of all refills dispensed in addition to new prescriptions may generate a pattern of medication-related problems unique from other approaches to monitoring. The types of medication-related problems identified by pharmacists delivering CoMM and the drug classes involved in problems have not been described, so this study seeks to fill that gap in the literature.

## 2. Materials and Methods

This pilot study of a single independent U.S. community pharmacy filling approximately 1600 prescriptions per week for 3000–3500 patients per year used the pharmacy’s dispensing and clinical records as its data source. Clinical records contain medication-related problems documented by the pharmacists. Patients filling at least one prescription at the pharmacy and having at least one medication-related problem identified by a pharmacist during a 12-month period were included. Patients were characterized by age, sex, and medications dispensed count. Medications dispensed count was the number of unique eight-digit level Generic Product Identifier codes dispensed by the pharmacy to the patient during the study period.

All pharmacists at the pharmacy perform CoMM for each dispensed medication. To assist pharmacists in their assessments, the clinical documentation system flags potential problems. Alerts for potential nonadherence are generated based on out of range proportion of days covered. Alerts for potential therapeutic duplication and drug-drug interactions are generated from the pharmacy dispensing system. High-risk medications for patients ≥65 years old, based on modified Beers criteria, are flagged. Other potential problems are identified by the pharmacists during their assessment. After assessing the involved medication and collecting any additional information needed from the patient, a pharmacist identifies the patient’s medication-related problems, takes action to resolve those problems, and documents a brief summary of each problem and action taken. All problems identified for study patients were included in this study. A medication-related problem appeared in the data each time it was documented by a pharmacist. Each appearance of a recurring problem contributed to the total number of medication-related problems. The pharmacists explicitly labeled 95% of problems as either nonadherence, therapeutic duplication, drug-drug interaction, high-risk medication for patient ≥65 years old, or new prescription needed due to rescheduling of controlled substance. The researchers labeled the remaining problems as one of these types or a sixth type, other medication-related problems. Examples of other medication-related problems include prescription of a medication to which the patient was potentially allergic and the need for additional therapy. The drug class for each medication involved in the medication-related problems was determined from the two-digit level Generic Product Identifier code. For therapeutic duplication and drug-drug interaction, drug classes for both medications were determined.

Patient characteristics of age, sex, and medications dispensed count were summarized for all study patients and four subgroups: <65 years old, ≥65 years old, <8 medications dispensed, and ≥8 medications dispensed. The distribution of the types of medication-related problems was produced for all patients and each subgroup. The drug classes involved in problems were characterized for each type of problem. The University of Iowa Institutional Review Board determined the project did not meet the regulatory definition of human subject research.

## 3. Results

A total of 8439 medication-related problems were identified for 1566 patients with at least one problem during a one-year period (Table 1). Fewer than 19% of the problems were recurrences of a previously documented problem. The pharmacy dispensed 82,507 prescriptions (new and refill) to the 1566 patients during the year, meaning one medication-related problem was identified on average every 9.78 prescriptions dispensed to these patients. Unique medications dispensed count ranged from 1–43 per patient with a median of eight. The largest proportion of medication-related problems was nonadherence (63.2%). The second largest proportion was therapeutic duplication (22.3%), followed by drug-drug interaction (6.3%). All subgroups except those dispensed <8 medications exhibited this pattern.

Over 30% of the medications involved in medication-related problems were from the central nervous system and analgesic class (Table 2). Five classes accounted for nearly 70% of medications with nonadherence. Underuse of medications in these classes was more common than overuse. The cardiovascular class was most often involved in therapeutic duplication, arising from use of multiple antihypertensive medications. The drug classes composing the ‘Other’ category for high-risk medications were the genitourinary and musculoskeletal & joint classes.

## 4. Discussion

During one year, community pharmacists engaged in CoMM identified 8439 medication-related problems. The five drug classes most commonly involved in problems were central nervous system and analgesic, cardiovascular, anti-infective, respiratory, and endocrine, aligning with a UK study examining medication-related problems identified by community pharmacists where the prescriber was contacted for resolution [4]. The most common problem identified through CoMM was nonadherence, echoing the experiences of community pharmacists in Japan providing monthly home visits or phone calls to patients over age 40 with chronic conditions [5]. These findings highlight the importance of monitoring refills in addition to new prescriptions in community pharmacies. Limiting attention to identifying problems with a single fill of a medication, such as after discharge from the hospital [6], fails to identify problems that develop during therapy such as nonadherence. The growth of medication synchronization and adoption of community pharmacy-level performance tracking in areas such as adherence has prompted greater involvement of pharmacists in medication adherence. CoMM can identify a variety of medication-related problems; it is another tool that pharmacists can use to fulfill their adherence management role. Nonadherence to cardiovascular and endocrine medication classes was commonly documented by pharmacists engaged in CoMM. These classes are included among medication classes targeted by community pharmacy performance measures [7]. The alignment of identified problems with performance measures suggests strategic implementation of measures may be valuable for directing pharmacists’ attention to medication-related problems of interest.

Five medication-related problems identified on average per patient in the current study exceeded the average of three problems per year in a study of medication management based on comprehensive medication reviews by pharmacists from an independent community pharmacy located near the pharmacy engaged in CoMM in this study [8]. Patients receiving medication management visited pharmacists providing the services 1.7 times per year on average, limiting opportunities for pharmacist-patient interaction and identification of medication-related problems. Patients receiving CoMM in the present study filled 10 unique medications on average during the year and filled some of those medications multiple times, generating frequent opportunities for pharmacists to monitor medications. The timely identification and resolution of medication-related problems in community pharmacies can be enhanced by provision of CoMM in addition to medication management.

Health plans and employers managing medication therapy for a population can review members’ prescription claims to identify medication-related problems and propose solutions to problems. Problems are more likely to be resolved when solutions are tailored to the patient. Information needed to tailor solutions, such as reasons for nonadherence, is typically absent from claims and must be collected from patients. CoMM promotes information gathering from patients to identify problems and tailor solutions. CoMM complements a claims-based approach to identifying and resolving medication-related problems.

The first limitation we acknowledge is the study was conducted in a single independent community pharmacy. The implementation of CoMM in other pharmacies may yield different results, though a comparable percentage of medication-related problems attributable to drug-drug interactions was found by community pharmacists identifying problems with new prescriptions and refills in pharmacies across Germany through an approach other than CoMM [9]. Because this study was a retrospective analysis, a second limitation is the data are limited to what was collected during the course of clinical care and not for the purposes of research. The categorization of medication-related problems does not align precisely with common categorizations in the literature [10,11]. The pharmacists did not document their actions as consistently as they documented the problems they identified. For this reason, this study focused on describing the medication-related problems documented by the pharmacists. A study of actions taken by pharmacists performing CoMM is planned.

## 5. Conclusions

Systematic monitoring of new prescriptions and refills by community pharmacists performing CoMM resulted in identification of medication-related problems that might otherwise have gone undetected. CoMM complements medication management performed by pharmacists, as well as claims-based reviews performed by health plans, and positions pharmacists to monitor the safety and effectiveness of medication therapy. Further study is needed to evaluate CoMM in other community pharmacies.

## Figures and Tables

**Table 1:** Patient characteristics and distribution of types of medication-related problems.

	Total	Age (Years)		Medications Dispensed	
		<65	≥65	<8	≥8
Patients					
*n*	1566	726 ^1^	838 ^1^	680 ^1^	872 ^1^
Mean age, years (SD)	63.5 (19.8)	47.0 (15.7)	77.8 (8.9)	59.8 (19.8)	66.6 (19.1)
≥65 years, *n* (%)	838 (53.5%)	0	838 (100%)	305 (44.9%)	530 (60.8%)
Female, *n* (%)	844 (53.9%)	366 (50.4%)	477 (56.9%)	340 (50.0%)	497 (57.0%)
Mean medications dispensed count (SD)	9.9 (6.6)	8.7 (6.2)	10.9 (6.7)	4.5 (1.8)	14.1 (5.8)
Mean medication-related problems per patient	5.4	4.5	6.2	2.5	7.7
Medication-related problems					
*n*	8439	3260 ^2^	5174 ^2^	1692 ^2^	6731 ^2^
Nonadherence	63.2%	68.3%	60.0%	71.9%	61.1%
Therapeutic duplication	22.3%	21.4%	22.9%	20.1%	22.9%
Drug–drug interaction	6.3%	4.5%	7.5%	1.2%	7.6%
Other	3.3%	3.3%	3.2%	3.4%	3.1%
High-risk medication for patient ≥65 years old	2.7%	0.1%	4.4%	1.7%	3.0%
New prescription needed due to rescheduling of controlled substance	2.2%	2.4%	2.1%	1.7%	2.3%

SD: standard deviation. ^1^ Patients with missing data on a selected variable are not included in the table. For example, there were two patients for whom no birthdate was recorded. ^2^ Medication-related problems for patients with missing data on a selected variable are not included in the table. For example, there were five medication-related problems in two patients for whom no birthdate was recorded.

**Table 2:** Frequency of drug classes involved in medication-related problems by type of medication-related problem.

	Total	Non-Adherence	Therapeutic Duplication	Drug-Drug Interaction	High-Risk Medication for Patient ≥65 Years Old	New Prescription Needed Due to Rescheduling of Controlled Substance
N	10,855 ^1^	5336 ^1^	3764 ^1^	1068 ^1^	230 ^1^	183 ^1^
Drug class						
CNS and Analgesic	30.1%	26.0%	34.5%	19.6%	49.1%	100%
Cardiovascular	21.9%	12.9%	38.8%	15.1%	11.7%	NA
Anti-Infective	11.4%	14.1%	6.9%	18.9%	NA	NA
Respiratory	6.2%	8.6%	4.5%	1.3%	10.9%	NA
Endocrine	5.6%	7.0%	5.1%	1.3%	1.3%	NA
Gastrointestinal	4.6%	6.7%	2.9%	0.9%	NA	NA
Dermatology	4.1%	6.1%	2.3%	1.6%	NA	NA
Nutrition and Hematology	3.8%	3.9%	1.5%	11.7%	NA	NA
Undocumented	2.7%	NA	NA	27.2%	NA	NA
Ophthalmic	2.2%	3.5%	1.0%	0.8%	2.6%	NA
Other	7.4%	11.2%	2.5%	1.6%	24.4%	NA

CNS: central nervous system; NA, not applicable. ^1^ Number of medications involved in medication-related problems.
